# Gene silencing based on RNA-guided catalytically inactive Cas9 (dCas9): a new tool for genetic engineering in *Leptospira*

**DOI:** 10.1038/s41598-018-37949-x

**Published:** 2019-02-12

**Authors:** L. G. V. Fernandes, L. P. Guaman, S. A. Vasconcellos, Marcos B. Heinemann, M. Picardeau, A. L. T. O. Nascimento

**Affiliations:** 1 Laboratório Especial de Desenvolvimento de Vacinas, Instituto Butantan, Avenida Vital Brasil, 1500, 05503-900 Sao Paulo, SP Brazil; 2 Universidad Tecnológica Equinoccial, Centro de Investigación Biomédica, Facultad de Ciencias de la Salud Eugenio Espejo, Avenida Mariscal Sucre y Mariana de Jesús. Campus Occidental, 170105 Quito, Ecuador; 3 Laboratório de Zoonoses Bacterianas do VPS, Faculdade de Medicina Veterinária e Zootecnia, USP, Avenida Prof. Dr. Orlando Marques de Paiva, 87, 05508-270 Sao Paulo, SP Brazil; 4 Institut Pasteur, Biology of Spirochetes Unit, 25 rue du Dr Roux, 75723 Paris, France

## Abstract

Leptospirosis is a worldwide zoonosis caused by pathogenic bacteria of the genus *Leptospira*, which also includes free-living saprophyte strains. Many aspects of leptospiral basic biology and virulence mechanisms remain unexplored mainly due to the lack of effective genetic tools available for these bacteria. Recently, the type II CRISPR/Cas system from *Streptococcus pyogenes* has been widely used as an efficient genome engineering tool in bacteria by inducing double-strand breaks (DSBs) in the desired genomic targets caused by an RNA-guided DNA endonuclease called Cas9, and the DSB repair associated machinery. In the present work, plasmids expressing heterologous *S. pyogenes* Cas9 in *L. biflexa* cells were generated, and the enzyme could be expressed with no apparent toxicity to leptospiral cells. However, *L. biflexa* cells were unable to repair RNA-guided Cas9-induced DSBs. Thus, we used a catalytically dead Cas9 (dCas9) to obtain gene silencing rather than disruption, in a strategy called CRISPR interference (CRISPRi). We demonstrated complete gene silencing in *L. biflexa* cells when both dCas9 and single-guide RNA (sgRNA) targeting the coding strand of the β-galactosidase gene were expressed simultaneously. Furthermore, when the system was applied for silencing the *dnaK* gene, no colonies were recovered, indicating that DnaK protein is essential in *Leptospira*. In addition, flagellar motor switch FliG gene silencing resulted in reduced bacterial motility. To the best of our knowledge, this is the first work applying the CRISPRi system in *Leptospira* and spirochetes in general, expanding the tools available for understanding leptospiral biology.

## Introduction

The genus *Leptospira* includes both pathogenic and saprophytic species^1,2^. Pathogenic *Leptospira* are the etiological agents of leptospirosis, while saprophytic bacteria are environmental free-living organisms. Humans are infected via contact with urine of wild or domestic animal carriers, either directly or indirectly through contaminated water or soil^3^.

Leptospires enter the host mainly via intact sodden or damaged skin or mucosa. The initial phase exhibits a wide range of nonspecific symptoms such as fever, chills, headache, and myalgia. Leptospirosis can progress to a severe condition known as Weil’s syndrome, corresponding to 5–15% of reported cases^1^. Another severe manifestation of the disease, leptospirosis-associated pulmonary hemorrhage syndrome, was first described in North Korea and China and has been increasingly reported worldwide^4,5^.

The mechanisms responsible for leptospirosis pathogenesis are still poorly understood, mainly due to the lack of genetic tools available for this pathogen. In the past decade, significant advances in the field have been made; Saint Girons *et al*.^6^ were able to engineer the LE1 prophage from *L. biflexa* into an *E. coli*-*L.biflexa* shuttle vector, only replicative in the saprophytic strains of *Leptospira*. A conjugative vector was also described^7^, and more recently, Pappas *et al*.^8^ engineered a shuttle-vector replicative in *E. coli* and all leptospiral strains, including pathogens, favoring the creation of recombinant *Leptospira* spp. and gain-of-function phenotype studies, especially in the saprophyte strain.

Current techniques of gene inactivation rely on homologous recombination for targeted genetic knockout or random transposon insertion^9–15^. However, the frequency of knockout mutants recovered for homologous recombination is extremely low, suggesting an inefficient capacity for double-recombination events. The transcription activator-like effectors (TALE) have also been used for specific gene silencing in both saprophytic and pathogenic strains, but this technique requires the synthesis of large and specific genes for each target^16^.

Recently, the CRISPR (clustered regularly interspaced short palindromic repeat) system has emerged as a powerful strategy for making mutants in both prokaryotic and eukaryotic cells^17,18^. This system has been found in the genomes of most Archaea and Bacteria, playing an important role in their immunity against phages and invading plasmids^19,20^.

To date, there are several CRISPR systems characterized, and among them, the *Streptococcus pyogenes* type II system has been the most explored. The requirement of only Cas9 nuclease for DNA cleavage and a single-guide RNA (sgRNA) for target specificity, has called the attention for the applicability of this system as a biotechnological tool^21,22^. The CRISPR/Cas9 tool has been applied in several prokaryotes^17,18,23^ and eukaryotes^24–29^.

The generation of DSBs by RNA-guided Cas9 cleavage must be repaired for cell viability. The distinct pathway used to repair induced DSBs dictates the type of genome editing: non-homologous end joining (NHEJ) and homology-directed repair (HDR)^30–32^.

Eukaryotes can repair DSBs introduced by Cas9 by directly ligating broken DNA ends using NHEJ in the absence of a repair template DNA molecule^33,34^. In contrast, Cas9 cleavage in the chromosome of most bacteria is reported to be lethal to the cells in the absence of a template for recombination^35,36^. This lethality could be overcome by the use of a variant of Cas9, lacking nuclease activity, called catalytically dead Cas9 (dCas9), capable of binding specific DNA targets and preventing gene transcription, resulting in gene silencing rather than disruption^37^. This strategy, called CRISPRi, has been successfully applied in various organisms^38–42^.

In this work, we report the application of *S. pyogenes* CRISPR/Cas9 system in saprophytic *L. biflexa* aiming to generate mutants. The rational to employ *L. biflexa* was based to the fact that *L. interrogans* are fastidious bacteria while the saprophyte strain has higher transformation efficiency and shorter incubation time for colony formation. We describe for the first time DSB lethality in *L. biflexa* after RNA-guided Cas9 cleavage and the successful application of dCas9 and sgRNA-driven gene silencing in leptospiral cells.

The genes selected in this work were β-galactosidase, *dnaK* and *fliG*. In the case of β-galactosidase, the selection followed the rational employed by Pappas *et al*.^16^: readiness in phenotype evaluation (chromogenic substrate) and its non-essential nature, allowing colony recovering in case of gene inactivation/silencing. DnaK and FliG proteins were chosen due high protein expression and possible decrease in bacterial motility, respectively.

We believe that the application of this technology in pathogenic strains by generating mutants could advance our understanding of the mechanisms of leptospiral infection.

## Material and Methods

### Bacterial strains, plasmids and media

*L. biflexa* serovar Patoc strain Patoc1 was cultured at 28 °C under aerobic conditions, in liquid EMJH medium (Difco, BD, Franklin Lakes, NJ) and 1.2% noble agar (Difco) solid EMJH medium supplemented with 10% (vol/vol) *Leptospira* enrichment EMJH (Difco)^43^. When necessary, spectinomycin was added at 50 µg/mL.

*E. coli* DH5α (Invitrogen) and *E. coli* strain π1^44^ were used for general cloning into pGKLep4^6^ and pMaOri^8^ plasmids, respectively. *E. coli* plasmids pCas and pTarget^17^ were utilized for Cas9 and sgRNA scaffold amplification, respectively, and were a kind gift from Dr. Luiziana Ferreira da Silva (Laboratory of Bioproducts, Institute of Biomedical Sciences, University of São Paulo). The pdCas9-M-G6 plasmid^45^ was used for dCas9 amplification and was purchased from Addgene (https://www.addgene.org).

### Evaluation of J23119, minimal borrelial *flaB* or *lipL32* promoter activity in *L. biflexa*

The activity of the consensus bacterial promoter J23119^46^, minimal borrelial *flaB*^47^ or hypothetical minimal *lipL32* promoter (including the possible −10 and −35 regions) were evaluated in *L. biflexa* by heterologous protein expression. Briefly, J23119 promoter (35 bp) was genetically fused to *lipL32* 5′UTR, CDS and transcription terminator, by PCR. Definition of 5′UTR was based on the results of TSS (transcription start site) defined by Zhukova *et al*.^48^. The first PCR was performed with primers J23LipL32 F1 and LipL32 R (Table 1), with *L. interrogans* serovar Copenhageni strain M20 genomic DNA. This PCR product was used as template for a second reaction containing primers J23LipL32 F2 and LipL32 R. The J23119 promoter nucleotide sequence was obtained from the BioBrick part BBa_J23119 (iGEM Registry of Standard Biological Parts, www.partsregistry.org). For minimal borrelial *flaB* promoter, the same 2-step PCR was used, first with primer flaBLip32 F1 and then with flaBLipL32 F2, where both reactions were with LipL32 R as reverse primer. Primers p32min F and LipL32 R were used for amplification of minimal *lipL32* promoter. Complete *lipL32* (–334-TSS) promoter was used as control, obtained by PCR with primers p32 F and LipL32 R. The final PCR products containing *SacI* and *XbaI* restriction sites were digested and ligated into the pMaOri vector. As control, to rule out the possibility of spurious transcription, *lipL32* CDS and 5′UTR were cloned without any promoter at their 5′ end (LipL32control F and LipL32 R, Table 1). Recombinant plasmids were used to transform *E. coli* π1and *L. biflexa* serovar Patoc by electroporation as previously described^49^. Heterologous LipL32 protein expression was assessed by Western blotting and probing with mouse polyclonal anti-LipL32 antiserum (1:3,000) and horseradish peroxidase (HRP)-conjugated anti-mouse IgG antibodies (1:10,000, Sigma).

**Table 1:** Sequences and restriction sites of the primers used in this study.

Primer	Sequence (5′ → 3′)	Restriction Site
pMaOri F	AGTGACACAGGAACACTTAACG	—
pMaOri R	TATATTCTGTCCACATTTGTGG	—
Cas9 F	ATCGGCGGCCGCGGTTTGCAGTCAGAGTAGAATAG	NotI
Cas9 R	ATCGTCTAGATTAAGAAATAATCTTCATCTA	XbaI
p32Cas9 F	GCATAGAGCGGCCGCGAACAAGAAAGAGTCAGAG	NotI
Cas9fusion	ATATCTAAGCCTATTGAGTATTTCTTATCCATAGACT CTCCTTAGTTAGG	—
Cas9seq1	ATATTAAACTAATTTCGGAG	—
Cas9seq2	GTAGATTCTACTGATAAAGC	—
Cas9seq3	TCAGCTTCAATGATTAAACG	—
Cas9seq4	TGAACGCATGACAAACTTTG	—
Cas9seq5	AATTGATTAATGGTATTAGG	—
Cas9seq6	ACAAAGTTTCCTTAAAGACG	—
Cas9seq7	TGGGGAAACTGGAGAAATTG	—
Cas9seq8	AATCAGTGAATTTTCTAAGC	—
Spc F	CGATTCAAACATTAAAAATCG	—
Spc R	CACTCACCGTATATAAATTCTC	—
J23LipL32 F1	GCTCAGTCCTAGGTATAATACTAGTCTAACTAAGGAG AGTCTATG	—
J23LipL32 F2	GCATAGAGAGCTCTTGACAGCTAGCTCAGTCCTAGGTA TAATACTAGTCTAAC	SacI
flaBLip32 F1	TTTTTTAATTTTTGTGCTATTCTTTTTAACCTAACTAAGG AGAGTCTATG	—
flaBLip32 F2	TAGAGAGCTCTTCTTTTTTTTTAATTTTTGTGCTATTCTT	SacI
p32min F	TAGAGAGCTCGATTTACAAAAAATTCCTAAG	SacI
p32 F	TAGAGAGCTCGAACAAGAAAGAGTCAGAG	SacI
LipL32 R	GCATAGATCTAGATATTCAAATGTAGTTTTAGG	XbaI
pMaOri2 F	ACGCAATGTATCGATACCGAC	—
pMaOri2 R	ATAGGTGAAGTAGGCCCACCC	—
p32XmaIF	atcgaCCCGGGGAACAAGAAAGAGTCAGAG	XmaI
Scaffold R	tcgaCCCGGGAAAAAAAGCACCGACTCGGTGCCACTTTTTCAAG	XmaI
sgRNAbgal1 F	CATACCGTGATTTTCAATTTGCCAACCTACAACCGGTTTTAGAGCTAGAA	—
sgRNAbgal2 F	CATACCGTGATTTTCGGGAACCACCCCGCGGTTGTGTTTTAGAGCTAGAA	—
sgRNAdnaK1 F	CATACCGTGATTTTCACACGAGCCGCAATTTCTTGGTTTTAGAGCTAGAA	—
sgRNAdnaK2 F	CATACCGTGATTTTCAGCCGGTGACGAATCAAAGAGTTTTAGAGCTAGAA	—
sgRNAfligF	CATACCGTGATTTTCAACCGCTACCAAAAAGATGGGTTTTAGAGCTAGAA	—
qPCRBgal F	ATTGTGACAGCAATGGCAAA	—
qPCRBgal R	GTGGGGAATAATCCACATCG	—
qPCR16S F	GGTGCAAGCGTTGTTCGG	—
qPCR16S R	GATATCTACGCATTTCACCGC	—

*Underlined are the restriction sites.

### Construction of Cas9-containing plasmids

The *Leptospira*-*E. coli* shuttle vector pMaOri^8^, replicative in saprophytic, intermediate and pathogenic strains, was used as backbone for *cas9* gene ligation. Two promoters directing *cas9* transcription were used: the native *cas9* promoter from *S. pyogenes* and the leptospiral complete *lipL32* gene promoter. pCas vector^17^ was used as template for *cas9* amplification with Platinum® Taq DNA Polymerase High Fidelity (Thermo). To obtain the native promoter plus *cas9* CDS, pCas plasmid was utilized as template with primers Cas9 F and Cas9 R (Table 1), containing *NotI* and *XbaI* restriction sites at the 5′ end for further ligation at pMaOri. The genetic fusion of *lipL32* promoter (p32) to the *cas9* CDS was achieved by the megaprimer strategy. First, the *lipL32* promoter was amplified from *L. interrogans* genomic DNA with primers p32Cas9 F and Cas9fusion (Table 1), the latter containing a complementary sequence to the end of the promoter and a 5′overhang with the first 30 nucleotides of *cas9* CDS; the amplicon was used as a forward primer along with pCas R to generate the fused gene, which was re-amplified with p32 F and Cas9 R. PCR products were digested with *NotI* and *XbaI* and ligated into the corresponding sites of pMaOri, transformed in *E. coli* π1 strain by heat shock and transformants were plated onto LB plates containing spectinomycin (50 µg/mL) and dT (0.3 mM). The plasmids were designated pMaOriCas9 (native promoter, pNative) and pMaOri.p32Cas9 (*lipL32* promoter) and clones with no mutation at *cas9* gene were used for further studies.

### Polyclonal antiserum production

*L. interrogans* serovar Copenhageni strain Fiocruz L1–130 genomic DNA was used to produce recombinant DnaK (LIC_RS02730, namely LIC10524) and FliG (LIC_RS00120, namely LIC10023). Recombinant proteins (10 µg) were used to immunize BALB/c mice for polyclonal antiserum production. Both antisera are able to recognize *L. biflexa* homologous proteins due to their high similarity. All animal studies were approved by the Ethical Committee for Animal Research of the Instituto Butantan, Brazil, registered under protocol no8543200617. The Committee for Animal Research in Instituto Butantan adopts the guidelines of the Brazilian College of Animal Experimentation (COBEA.)

### Validation of Cas9 expression in recombinant *E. coli* and *L. biflexa*

Recombinant *E. coli* and *L. biflexa* containing pMaOri, pMaOriCas9 or pMaOri.p32Cas9 were recovered from the medium by centrifugation (6,000 rpm, 15 min), washed twice in PBS and resuspended in the same buffer. Final bacterial suspensions were diluted and normalized to an absorbance of 10 at 420 nm. Samples were then mixed with denaturing buffer (10% SDS, 50% glycerol, 0.5% bromophenol blue, 3% β-mercaptoethanol) and heated for 10 min at 96 °C, and proteins in 20 μL of sample were separated by 7.5% SDS-polyacrylamide gel electrophoresis (SDS-PAGE) for 2 h. Proteins were transferred onto nitrocellulose membranes (Hybond ECL; GE Healthcare) on semidry equipment, and transfer efficiency was assessed by Ponceau S staining (Sigma). Membranes were blocked with 10% nonfat dry milk in PBS containing 0.05% Tween 20 (PBS-T) and then incubated with mouse monoclonal anti-Cas9 antibodies (SAB4200701, 1:10,000 in PBS-T containing 1% milk, Sigma) for 1 h at room temperature. After washing, detection of bound antibodies was performed by incubation with HRP-conjugated anti-mouse IgG (1:10,000, Sigma) for 1 h. Incubation with polyclonal anti-DnaK (1:1,000) was used as constitutive and loading control for leptospiral cell extracts. Protein reactivity was revealed by using SuperSignal^TM^ West Dura Extended Duration Substrate (Thermo Scientific) and the luminescence generated was detected with the aid of an Amersham Imager 600 (GE).

### Assessment of Cas9 nuclease activity in *E. coli*

Competent *E. coli* cells containing the plasmid pMaOriCas9 or pMaOri.p32Cas9 or wild-type strain cells were transformed with 10 ng of plasmid pGKLep4 or pGKLep4 containing the J23119 promoter directing the transcription of sgRNA targeting the *E. coli* β-galactosidase gene (b0344) (pGKLep4sgRNAbgalEcoli) or leptospiral β-galactosidase (LEPBI_RS00135), as control. After transformation, cells were seeded on LB plates supplemented with both spectinomycin (50 μg/mL, selection of pMaOri backbone plasmids) and kanamycin (50 μg/mL, selection of pGKLep4 backbone plasmids). Wild-type cells transformed with pGKLep4 plasmid were seeded on LB plus kanamycin plates.

### Selection of protospacer, constructions of sgRNA cassettes and plasmid ligation

Desired gene sequences were obtained from GenBank (https://www.ncbi.nlm.nih.gov/genbank/) and submitted to the webserver CHOPCHOP (http://chopchop.cbu.uib.no/)^50^, with the basic input comprising CRISPR/Cas9 and PAM (protospacer adjacent motif) NGG. Protospacers were chosen on the basis of their score and proximity to the N-terminus of the resulting proteins. The selected protospacers, β-galactosidase, *dnaK* and *fliG*, are shown in Table 2. Protospacer transcription along with sgRNA scaffold for Cas9 recognition was driven by the leptospiral *lipL32* promoter, the same sequence used for Cas9 expression on pMaOri.p32Cas9. First, sgRNA scaffold sequence contained in the pTarget vector^17^ was amplified by PCR with primers sgRNAF (depending on the protospacer) and Scaffold R. At the 5′ end of each forward primer, a 20 nt sequence regarding the selected protospacer sequence was included, along with a sequence complementary to the end of the *lipL32* promoter. Amplicons were purified and used as reverse megaprimer in a second PCR mixture containing p32XmaI F as forward primer. Fused amplicon was purified, digested with XmaI restriction enzyme and then ligated to pMaOriCas9 or pMaOri.p32Cas9 vectors previously digested with the same enzyme. Ligation reactions were used to transform *E. coli* π1 cells, which were seeded on LB plus spectinomycin and dT (0.3 mM). Final recombinant plasmids were confirmed by colony PCR (pMaOri2 F and pMaOri2 R) and sequencing. *L. biflexa* cells were grown in liquid EMJH and then transformed by electroporation with recombinant pMaOriCas9 or pMaOri.p32Cas9 containing different sgRNA.

**Table 2:** Protospacers selected for gene targeting.

sgRNA	Protospacer sequence	Pairing to
sgRNAbgal1	AATTTGCCAACCTACAACCG	Coding strand
sgRNAbgal2	GGGAACCACCCCGCGGTTGT	Template strand
sgRNAdnaK1	ACACGAGCCGCAATTTCTTG	Coding strand
sgRNAdnaK2	AGCCGGTGACGAATCAAAGA	Template strand
sgRNAfliG	AACCGCTACCAAAAAGATGG	Coding strand

### Construction of dCas9-expressing plasmids

The pdCas9-M-G6 plasmid^45^ was used as template in PCR mixture containing Cas9 F and Cas9 R (Table 1), with *Not*I and *Xba*I restriction sites at the 5′ end for later ligation to the pMaOri vector. PCR conditions and ligation parameters were the same as described above for pMaOriCas9 construction. After confirmation of recombinant plasmid, pMaOri.dCas9 was digested with *Xma*I for inclusion of sgRNA cassettes. Plasmids containing sgRNA sequence alone were also constructed and employed as control for gene silencing. Briefly, sgRNA cassettes were amplified, digested with *Kpn*I and *Bam*HI enzymes and ligated at the same restriction sites in the pGKLep4 plasmid.

### Validation of β-galactosidase gene silencing in *L. biflexa*

Confirmation of genetic knockdown was performed by β-galactosidase colorimetric assay, according to the methods described by Pappas and Picardeau^16^, with slight modifications. Briefly, *L. biflexa* cultures were counted by using a Petroff-Hausser counting chamber and a total of 3 × 10^8^ cells per sample were centrifuged at 4,000 × g for 15 min. The supernatant was discarded and cells resuspended in 1 mL of Z buffer (pH 7, containing 130 mM monosodium phosphate, 40 mM disodium phosphate, 10 mM potassium chloride, 2 mM magnesium sulfate and 40 mM β-mercaptoethanol). The absorbance of each sample was measured at 420 nm, and then, 40 µL of chloroform and 30 µL of 0.1% SDS were added. Samples were vortexed and then allowed to stand at 30 °C for 5 min. Right after incubation, 200 µL of 13.27 mM *o*-nitrophenyl-*β*-*D*-galactopyranoside (ONPG, Sigma) in Z buffer were added to the samples. After development, the reaction was stopped by mixing 250 µL of sample with 100 µL of 1 M sodium carbonate. The color change was evaluated using a spectrophotometer to measure absorbance at 420 nm. Also, X-gal chromogenic substrate was used to assay for β-galactosidase activity. Briefly, 5 × 10^8^ leptospiral cells were resuspended in 300 µL of PBS containing 5 mg/mL X-gal, and the blue color was determined by spectrophotometer readings at 595 nm.

### RNA extraction and qPCR

Leptospiral cells in late-log phase in 30 mL EMJH medium were collected by centrifugation (6,000 rpm for 15 min) in RNase-free tubes. The resulting pellet was resuspended in 1 mL of Trizol reagent (Invitrogen) and RNA was purified according to the manufacturer’s instructions. cDNA was synthesized using 2 µg total RNA, the SuperScript III reverse transcriptase kit (Invitrogen) and random hexamers, according to standard protocols. RT-qPCR was performed in a reaction volume of 20 μL containing 1 µL of cDNA, 400 nM of each oligonucleotide and 10 μL of SYBR Green PCR Master Mix (Applied Biosystems), as recommended by the manufacturer. All reactions were performed in triplicate in 96-well optical plates. Negative controls using all the reagents but cDNA were used (NTC, no template control). Cycling conditions were: 50 °C for 2 min and 95 °C for 10 min, followed by 40 cycles of 95 °C for 15 s and 60 °C for 30 s. The relative gene expression among the *L. biflexa* cells containing different plasmids was determined using the 2−ΔΔCT method. The constitutive 16 S gene was used as the internal normalization control.

### Phenotype stability experiments

Gene silencing stability was performed as follows: *L. biflexa* cells containing pMaOri.dCas9sgRNAbgal1 grown in EMJH plus spectinomycin were transferred to new EMJH liquid medium (1:10 dilution) with no antibiotic addition. Serial passages were performed and at each passage, β-galactosidase activity was measured, as described above, and compared to that exhibited by leptospires grown in EMJH plus antibiotic (100% gene silencing). Confirmation of plasmid maintenance was performed by PCR with pMaOri2 primer.

## Results

### Assessment of different promoter activity in *L. biflexa*

**Figure 1:**
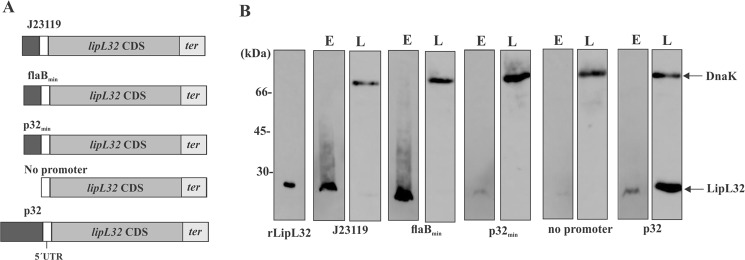
Assessment of promoter activity in *L. biflexa*. (**A**) *L. interrogans lipL32* gene CDS, 5′UTR plus transcription terminator were genetically fused to different promoters. *L. biflexa* cell were electroporated with the different recombinant plasmids and promoter activity was indirectly accessed by probing whole cell extracts with anti-LipL32 antiserum for detecting heterologous LipL32 expression (**B**). Anti-DnaK antiserum was employed as a loading control. LipL32 expression was assayed in both *E. coli* (E) and *L. biflexa* (L). As a negative control, a construction in which no promoter was added to rule out the possibility of spurious DNA transcription. The *E. coli* and *L. biflexa* protein blottings are from different gels (individually depicted in Fig. S1). For comparative purposes, the lanes were cut and organized by promoters.

We evaluated different minimal promoters to determine which one is efficiently recognized by *L. biflexa* transcriptional apparatus. Figure 1A describes the different constructions used; the consensus *E. coli* promoter J23119 (35 nt)^46^, the previously characterized minimal borrelial *flaB* promoter (35 nt)^47^ and a hypothetical minimal *lipL32* promoter (35 nt), comprising the -10 and -35 sequences, were used. As positive control, the complete *lipL32* promoter (334 nt) was utilized, and to rule out the possibility of spurious transcription in the plasmid used, the *lipL32* gene was also cloned without any promoter sequence at its 5′ end. Inserts were ligated into pMaOri and recombinant plasmids were used to transform *L. biflexa*; transcriptional activity of different promoters was assessed indirectly by heterologous LipL32 protein detection.

After Western blotting, strong LipL32 reactivity was obtained only with the complete *lipL32* promoter, denoting that leptospires were not effective in recognizing the minimal promoters tested (Fig. 1B and Fig. S1). Accordingly, no band was detected when no promoter sequence was included, indicating that no spurious transcription occurred in pMaOri. DnaK protein was utilized as a loading control.

On the other hand, strong LipL32 signal was detected in cell extracts from *E. coli* π1 cells containing the constructs in which J23119 and *flaB*_min_ promoters were used, followed by a modest protein signal when leptospiral *lipL*32 promoter was used. These results reinforced the notion that leptospiral cells were indeed defective in recognizing these minimal promoters, and therefore, only the complete *lipL32* promoter (p32) was selected for further use.

### Heterologous Cas9 expression in *L. biflexa* and nuclease activity validation in *E. coli*

**Figure 2:**
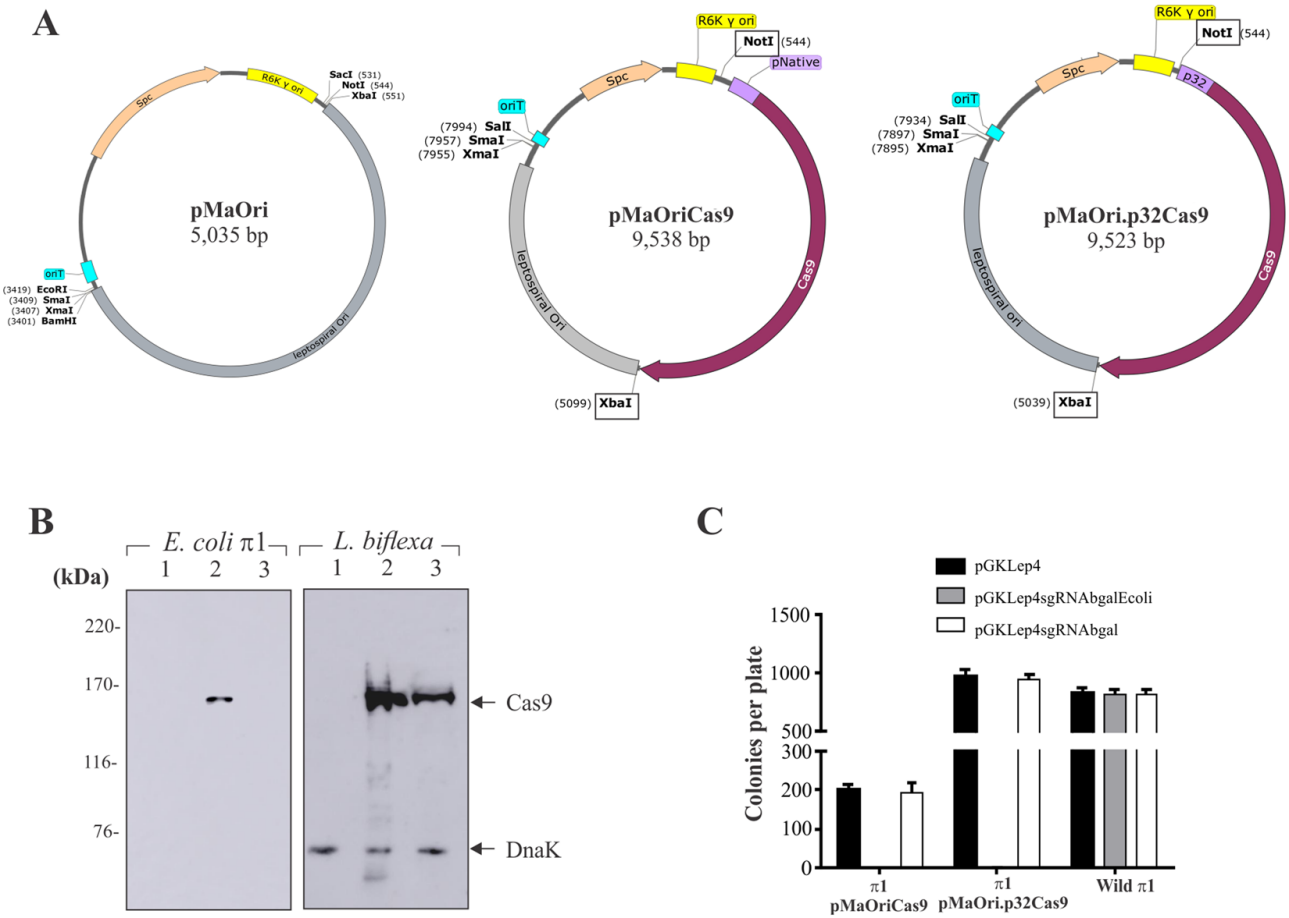
Heterologous expression of Cas9 in *L. biflexa* cells and nuclease activity validation in *E. coli*. (**A**) Map of the plasmids obtained in this work, depicting important features and unique restriction sites. (**B**) Imunoblots of *E. coli* and *L. biflexa* containing wild type pMaOri (1), pMaOriCas9 (2) or pMaOri.p32Cas9 (3) with anti-Cas9 and anti-DnaK antibodies. Data of *E. coli* and *L. biflexa* come from different blottings. (**C**) Transformation efficiency of *E. coli* cells containing plasmids pMaOriCas9, pMaOri.p32Cas9 or no plasmid with plasmids pGKLep4 (wild type) or pGKLep4 containing J23119 promoter directing the transcription of sgRNA targeting the *E. coli* βgal gene (pGKLep4sgRNAbgalEcoli) or an unrelated sequence (leptospiral β-galactosidase, pGKLep4sgRNAbgal), as control. After transformation, cells were seeded onto LB plates supplemented with spectinomycin and kanamycin. As a control, wild type cells transformed with pGKLep4 plasmids were seed in LB plus kanamycin plates. Cell counting are presented as average plus standard deviation of 3 independent experiments.

The schematic representation of the plasmids constructed in this work along with important features and unique restriction sites are presented in Fig. 2A. Due to the pMaOri backbone, both plasmids pMaOriCas9 and pMaOri.p32 Cas9 could be used to transform *Leptospira* spp. by electroporation or conjugation.

Cas9 expression in *E. coli* was only detected in the cells containing the pMaOriCas9 plasmid (Fig. 2B, *E. coli*, lane 2), leading us to conclude that these bacteria could recognize the *S. pyogenes* promoter, as expected^17^, but were not very effective in recognizing the leptospiral *lipL32* promoter (lane 3). Surprisingly, *L. biflexa* cells could recognize both promoters, because Cas9 expression was detected in recombinant bacteria containing pMaOriCas9 (Fig. 2B, *L. biflexa*, lane 2) and pMaOri.p32 Cas9 (lane 3), in similar fashion. DnaK protein was employed as a loading control. It is worth noting that no significant shift in the growth curve was observed when comparing Cas9-expressing cells to leptospiral cells containing empty pMaOri vector (data not shown).

To confirm whether the *cas9* genes ligated into the pMaOri backbone were indeed mutation-free and the corresponding protein functional and able to cleave DNA, *E. coli* cells containing pMaOriCas9 or pMaOri.p32Cas9 (spectinomycin resistance) were made competent and then transformed with a second plasmid, pGKLep4, empty or containing an expression cassette for sgRNA against β-galactosidase of *E. coli* or *L. biflexa*, used as negative control; sgRNA transcription was driven by the J23119 promoter. When *E. coli* containing either pMaOriCas9 or pMaOri.p32Cas9 were transformed with empty pGKLep4 or pGKLep4sgRNAbgal (targeting the leptospiral gene, kanamycin resistance), a large number of colonies were isolated on plates with double antibiotic selection (Fig. 2C). The discrepancy in absolute number regarding the pMaOriCas9 and pMaOri.p32Cas9 groups was possibly due to competence differences. Accordingly, when we used pGKLep4sgRNAbgalEcoli, no colonies were observed (Fig. 2C), indicating that the Cas9 protein retained nuclease activity and was able to induce DSBs in the *E. coli* genome, causing a lethal phenotype^35^. These results suggested that Cas9 expressed by both vectors is functional and able to cleave genomic DNA, and although no Cas9 band was detected by Western blotting in *E. coli* harboring pMaOri.p32Cas9 (Fig. 2B, *E. coli*, lane 3), it is likely that this protein was present in low copy number, possibly due to low *lipL32* promoter activity in *E. coli*, as observed in Fig. 1B.

### Cas9 and sgRNA co-expression is lethal to *L. biflexa*

**Figure 3:**
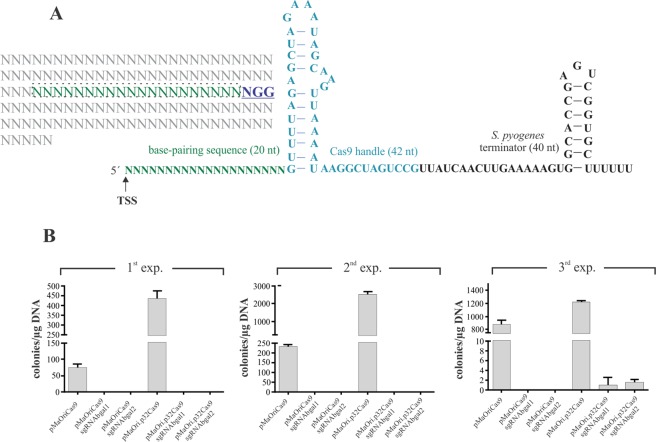
sgRNA-directed Ca9 genomic cleavage is lethal to *L. biflexa* cells. (**A**) Protospacers are selected based on the presence of a 3′ NGG (PAM). Schematic representation of sgRNA sequence is also shown; the first 20–25 nucleotides, starting from the transcription start site (TSS) are variable according to the gene target, followed by a sequence folded in a hairpin-like structure responsible for Cas9 interaction. Transcription termination is dictated by the presence of a *S. pyogenes* intrinsic terminator. (**B**) pMaOriCas9, pMaOri.p32Cas9 plasmids, alone or containing different sgRNA targeting the β-galactosidase gene were used to electroporate competent *L. biflexa* cells, which were seeded onto EMJH plates plus spectinomycin and grown colonies were counted and normalized per µg of DNA. Different experiments are presented and for each, cell counting is presented as average plus standard deviation of 3 different plates.

Protospacer to target the non-essential β-galactosidase gene in *L. biflexa* was selected by the CHOPCHOP^50^ webserver. Briefly, 20-mer sequences containing a 3′PAM, in this case NGG for *S. pyogenes* Cas9, were selected and transcribed along with a scaffold sequence containing a Cas9 handle and an intrinsic transcription terminator (Fig. 3A). The first 20 nucleotides of the sgRNA determine target specificity by Watson-Crick base pairing, and therefore, are easily editable.

Wild-type *L. biflexa* cells were transformed with pMaOriCas9 or pMaOri.p32Cas9 plasmids containing the sgRNA constructs. Transcription of the single-guided RNA targeting the *L. biflexa* β-galactosidase was driven by the strong leptospiral *lipL32* gene promoter. The plasmids without the sgRNA sequences were used for control.

The data obtained from three independent experiments showed that, even with different transformation rates (ranging from 75 to 880 and 436 to 2530 per µg DNA for pMaOriCas9 and pMaOri.p32Cas9, respectively), a pattern was clearly observed: no or very few colonies were obtained when both Cas9 and sgRNA were expressed (Fig. 3B), indicating that DSBs were lethal for *L. biflexa*, as observed for most prokaryotes, including *E. coli*^36^. Also, these results indicated that the protospacer sequences selected for sgRNA composition could successfully direct the Cas9 nuclease to the desired target sites.

Accordingly, the few colonies obtained in the third experiments after transformation with pMaOri.p32Cas9 containing sgRNAbgal1 or 2 were evaluated regarding Cas9 expression by Western blotting and sgRNA cassette presence by PCR with pMaOri2 F and R (Table 1). Our results indicated that neither Cas9 nor sgRNA were present in these bacteria (data not shown), suggesting that those colonies were spontaneous mutants resistant to spectinomycin. Indeed, these colonies, when investigated, displayed no decrease in β-galactosidase activity (data not shown).

### Development of a gene knockdown strategy using dCas9 and sgRNA

**Figure 4:**
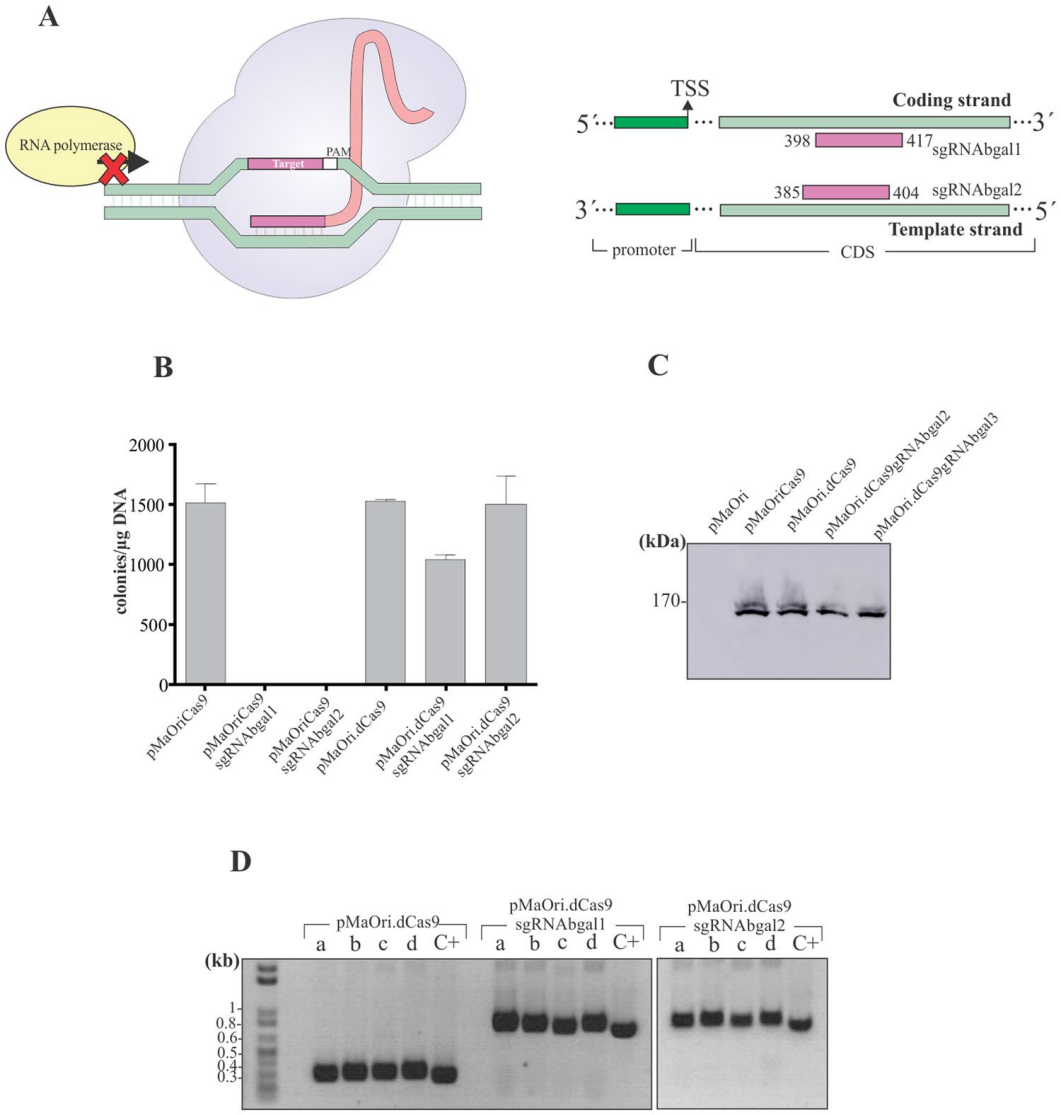
dCas9 and sgRNA simultaneous expression is tolerated by *L. biflexa* cells. dCas9, a nuclease-depleted variant of Cas9, when directed by a sgRNA, is capable of binding to specific target sequences, causing gene silencing rather than disruption due to a steric blockage of RNA polymerases elongation. sgRNA pairing to both coding (sgRNAbgal1) and template (sgRNAbgal2) strands were constructed (**A**). pMaoriCas9 and pMaOridCas9, alone or containing distinct sgRNA, were used to electroporate *L. biflexa* cells. Colonies were counted (**B**) and screened for dCas9 expression, by western blotting with monoclonal anti-Cas9 antibodies (**C**). Different colonies (a-d) were also assayed for sgRNA presence by PCR using flanking primers; (**D**) purified plasmids were employed as positive control (C+).

As DSBs were shown to be lethal to *L. biflexa* cells, a catalytically inactive (“dead”) Cas9 (dCas9) was used. This system, in which the Cas9 enzyme has two silencing mutations in nuclease domains, is utilized to target gene regulation rather than disruption, by blocking RNA polymerase from promoter recognition or elongation^37^ (Fig. 4A). Since the sgRNA used in this work are able to pair within the β-galactosidase (LEPBI_RS00135) CDS (sgRNAbgal1 and sgRNAbgal2 pairing to the coding and template strand, respectively), a blocking of RNA polymerase movement is expected.

*L. biflexa* wild-type cells were transformed by electroporation with pMaOri.dCas9 alone or containing sgRNAbgal1 or sgRNAbgal2; pMaOriCas9 alone or containing sgRNA were also utilized. As expected, no colonies were observed when wild-type Cas9 enzyme was expressed along with sgRNAs, corroborating DSB lethality. Accordingly, when dCas9 enzyme was expressed along with sgRNAs, a large number of bacteria were recovered (over 1000 colonies per µg DNA), comparable to the plasmids expressing only Cas9 or dCas9 (Fig. 4B).

To confirm that indeed the cells were harboring the plasmids containing both dCas9 and sgRNA (pMaOri.dCas9sgRNAbgal), different colonies (a-d) were selected from each plate and assessed for dCas9 expression by Western blotting and probing pooled total cell extracts with anti-Cas9 antibodies (Fig. 4C) and for the presence of the sgRNA cassette by PCR using pMaOri2 primers (Fig. 4D).

### Validation of β-galactosidase gene silencing

**Figure 5:**
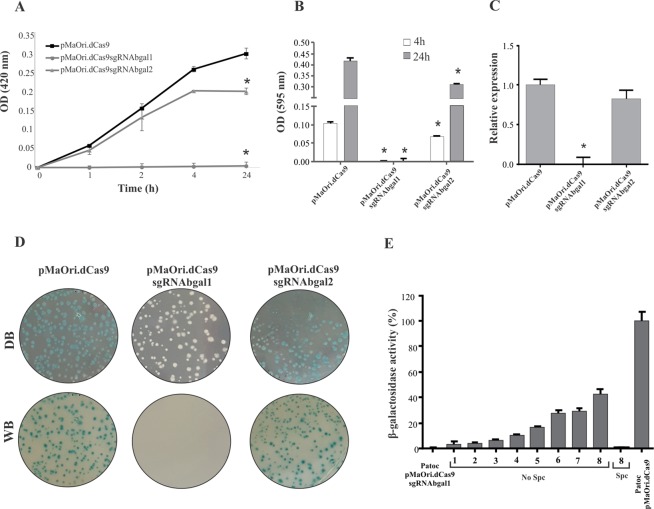
Total β-galactosidase gene silencing obtained by dCas9 expression along with sgRNA pairing to the coding strand. Different colonies were selected from the EMJH plates, grown in liquid media and then evaluated regarding β-galactosidase activity in a time-basis, employing ONPG substrate (**A**) and X-gal (**B**). Graphics show the average of densitometric reading plus standard deviation of 4 biological replicates. For statistical analysis, absorbance values displayed by cells containing dCas9 and sgRNA were compared to those displayed by cells containing pMaOri.dCas9 plasmid alone by Student’s two-tailed t test (*P < 0.05). (**C**) Relative transcription level of β-galactosidase gene in knockdown strains by qPCR. Presented values represent the mean plus standard deviation measurements. For statistical analyses, the absorbance or relative expression values displayed by the knockdown mutants were compared to those presented by the cells containing pMaOri.dCas9 alone (*P < 0.05). (**D**) X-gal solution was spread onto EMJH plates containing grown colonies, containing either pMaOridCas9 or this plasmid with sgRNA cassettes. Figures are presented against dark (DB) and white (WB) background. (**E**) *L. biflexa* cells containing pMaOri.dCas9sgRNAbgal1 grown in EMJH plus spectinomycin were transferred to new EMJH liquid medium (1:10 dilution) with no antibiotic (No Spc). Serial passages were performed (1–8) and at each passage, β-galactosidase activity was measured. The activity displayed by cells containing pMaOri.dCas9 plasmid was considered 100%.

Having recovered leptospiral cells transformed with plasmids containing both dCas9 and sgRNA, we decided to follow up and confirm whether gene silencing occurred: different colonies were isolated from plates and grown in liquid EMJH plus spectinomycin, and cells were evaluated regarding β-galactosidase activity by using two different chromogenic substrates, ONPG and X-gal. Enzyme activity was evaluated on a time basis, and when ONPG was utilized, there was a slight decrease (around 33%) in leptospiral cells when dCas9 was expressed along with sgRNAbgal2 compared to the cells expressing dCas9 alone (Fig. 5A). On the other hand, a complete gene silencing was observed when sgRNA was designed to hybridize to the coding strand (sgRNAbgal1), even when the reaction proceeded for 24 h, indicating a 100% reduction of gene transcription (Fig. 5A).

Gene silencing was also confirmed when X-gal substrate was used (Fig. 5B), with cells displaying reductions comparable to the previous experiment, corroborating the successful effect of sgRNAbgal1 on gene silencing. The major gene silencing effect of sgRNA targeting the coding strand was previously shown in *Staphylococcus aureus*^38^, indicating that our newly described system is feasible in *L. biflexa*.

Since the repression of gene expression is thought to occur at the transcriptional level, that is, sgRNA-directed dCas9 bound to DNA blocks RNA polymerase extension, we aimed to detect β-galactosidase mRNA levels in knockdown and control cells by qPCR. Consistent with the enzyme assays, bacteria expressing sgRNAbgal1 displayed a marked decrease in β-galactosidase mRNA levels, followed by a modest reduction in cells expressing sgRNAbgal2, compared to those expressing only dCas9 (Fig. 5C).

To rule out the possibility of gene silencing due to RNA-RNA duplex rather than CRISPRi, *L. biflexa* cells were transformed with plasmids containing sgRNA sequence alone and the recovered colonies display no reduction upon β-galactosidase activity (Fig. S2), confirming that dCas9 is necessary for gene silencing.

To reinforce the successful application of the system, the whole colony population grown in EMJH plus spectinomycin was evaluated. Briefly, 100 µL of 20 mg/mL X-gal solution were spread on the plates, which were then incubated at 30 °C for 24 h. Colonies containing pMaOri.dCas9 or pMaOri.dCas9sgRNAbgal2 showed a strong blue color (Fig. 5D, DB, dark background, WB, white background), corresponding to β-galactosidase activity detected in those cells (Fig. 5A,B); even though sgRNAbgal2 could reduce enzyme activity slightly, the qualitative nature of these experiments did not allow us to determine any measurable decrease. In contrast, all colonies containing pMaOri.dCas9sgRNAbgal1 appeared white (Fig. 5D), corroborating the complete gene silencing in our system.

When phenotype stability was evaluated, even after 8 passages in EMJH medium with no antibiotic selection, gene silencing could still be observed (Fig. 5E), denoting plasmid stability in leptospiral population, as previously shown^8^, which was confirmed by PCR assay (data not shown); accordingly, addition of spectinomycin in EMJH medium at the eighth passage completely restored full gene silencing by selecting plasmid-containing leptospires (Fig. 5E).

### *DnaK* gene silencing prevents *L. biflexa* growth in plates

**Figure 6:**
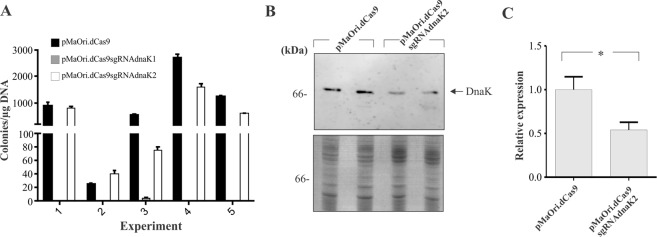
sgRNA targeting the coding strand of *dnaK* gene prevents leptospiral growth in plates. *L. biflexa* cells were transformed with the plasmid pMaOri.dCas9 alone or containing sgRNA targeting the coding (sgRNAdnaK1) or template (sgRNAdnaK2) strand of the *dnaK* gene. Colonies were counted (**A**) and DnaK protein levels were evaluated by western blotting probing cell extracts with anti-DnaK (**B**, upper panel**)**. Coomassie blue staining (B, lower panel) was used as loading control. (**C**) Relative transcription level of *dnaK* gene in knockdown strain by qPCR. Values are presented as average plus standard deviation of 3 replicates. For statistical analyses, relative expression values displayed by the knockdown mutant were compared to those presented by the cells containing pMaOri.dCas9 alone (*P < 0.05).

*L. biflexa* cells were transformed with the plasmid pMaOri.dCas9 alone or containing sgRNA targeting the coding (sgRNAdnaK1) or template (sgRNAdnaK2) strand of the *dnaK* (LEPBI_RS16560) gene CDS. After five independent experiments, despite the variation at transformation rates, no cells containing dCas9 and sgRNAdnaK1 could be recovered, even though a large number of colonies were observed in plates with pMaOri.dCas9 alone or containing the sgRNA hybridized to the template strand (sgRNAdnaK2) (Fig. 6A). It is worth mentioning that the few colonies obtained after transformation with pMaOri.dCas9sgRNAdnaK1 in experiment number three, when assayed by PCR, were negative for Cas9 CDS and sgRNA cassette (data not shown). When the protein (Fig. 6B) and mRNA levels (Fig. 6C) were assessed in the recovered leptospiral cells containing pMaOri.dCas9sgRNAdnaK2 in comparison to pMaOri.dCas9 alone, a slight reduction was observed. Despite this reduction in DnaK expression, no difference in growth curves was observed (data not shown).

### Flagellar motor switch *fliG* gene silencing reduces bacterial motility in plate

**Figure 7:**
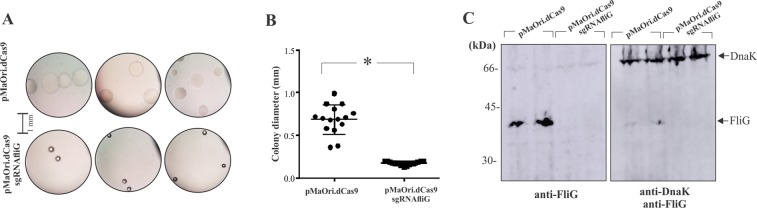
Flagellar motor switch FliG silencing reduces bacterial motility. (**A**) *L. biflexa* colonies containing wither pMaOri.dCas9 or pMaOri.dCas9sgRNAfliG were visualized under light microscope and their diameter calculated. (**B**) Graphic shows the average ± standard deviation of 15 colonies of each plate. For statistical analysis, diameter values displayed by cells containing dCas9 and sgRNA were compared to the ones displayed by cells containing pMaOri.dCas9 plasmid alone by Student’s two-tailed t test (*P < 0.05). (**C**) Different colonies were selected, grown in liquid media and cell extracts of two distinct leptospiral pools were transferred onto nitrocellulose membrane, which was probed with mouse anti-recombinant FliG (1:500) and HRP-conjugated anti-mouse IgG (1:5,000); after development with chemiluminescent substrate, the same membrane was washed and incubate with mouse anti-DnaK antiserum (1:3,000) as loading control.

A sgRNA cassette was constructed aiming hybridization with the coding strand of the *fliG* gene (LEPBI_RS16770) and ligated into pMaOri.dCas9 plasmid. *L. biflexa* cells were transformed with either pMaOri.dCas9 or pMaOri.dCas9sgRNAflig. The time required for the appearance of colonies of the latter group was considerably longer (~14 days vs 8 days). Similar number of colonies were obtained per µg of DNA employed (not shown) and a clear phenotype could be observed regarding their morphology, where smaller colonies were observed when dCas9 and sgRNA were expressed (Fig. 7A). After 18 days growth, distinct colonies were visualized at light microscope and diameter were calculated: bacteria transformed with pMaOri.dCas9 alone presented an average 0.69 ± 0.18 mm diameter, in contrast to 0.17 ± 0.02 mm calculated for colonies containing pMaOri.dCas9sgRNAflig (Fig. 7B), denoting deficiency in motility. Longer time required for growth and smaller colonies were also observed in *fliG2* (BB_0290) mutants in the spirochete *Borrelia burgdorferi*^51^.

Colonies were selected for growth in liquid media (no difference in growth curve, data not shown) and recovered bacteria were assayed regarding FliG protein expression, probing cell extracts with mouse anti-recombinant FliG. The phenotype observed was correlated with an abolished protein expression (Fig. 7C), indicating that FliG is necessary for bacterial motility. The data strengthened the importance of the system to studying leptospiral biology.

## Discussion

A pivotal step for understanding the basic biology and virulence of microorganisms is the ability to generate genetic mutations to assess the resulting phenotype^18^. Despite the recent advances in genetic tools for functional genomic analysis in many microbial species, target mutations or gene silencing in some microorganisms remains difficult to implement.

Genetic manipulations in *Leptospira* spp. have expanded in the past years, but are still limited. Random transposon insertion libraries have been performed in both pathogenic^9^ and saprophyte strains^10^. Although these studies have shed light on various gene functions and their role in pathogenesis and basic biology^52–58^, screening libraries of mutants is laborious, and in most cases, there is no evidence of the expected phenotype, since the products of several leptospiral genes are assigned as hypothetical proteins^59,60^. Moreover, there is no guarantee that the gene of interest will be knocked out as it is not possible to fully saturate the genome with all possible transposon insertions^9^.

The ability to knockout specific target genes in *Leptospira* was achieved by the use of homologous recombination, by using suicide plasmids containing an antibiotic resistance cassette flanked by homology arms^13^. However, as the recombination events are not induced by DSBs, the recovery rate of actual mutants is extremely low, and in most cases, only a single recombinant event is observed, culminating in whole plasmid insertion.

Pappas and Picardeau^16^ made the first attempt to control gene expression in *L. biflexa*, targeting the β-galactosidase gene by transcription activator-like effectors (TALEs). TALEs are a group of repressors that can modulate transcriptional activity by directly binding to a targeted sequence within the host genome^61^. Although encouraging results were obtained, this tool requires the design of one protein for each gene target, making this technique costly and laborious^62^.

The prokaryote immunity type II CRISPR/Cas system is emerging as a powerful biotechnological tool for manipulating the genome in various organisms. The Cas9 enzyme is an RNA-guided DNA endonuclease that can be easily programmed to target genomic sites depending on the interaction with the guide RNA sequence^63^.

Plasmids expressing heterologous *S. pyogenes* Cas9 in *L. biflexa* cells were generated, and the enzyme could be expressed with no apparent toxicity to leptospiral cells. Since Cas9 must be directed to its target sequence for genomic DSBs, we included different sgRNA in the Cas9-expressing plasmids for a one-step mutagenesis methodology. The β-galactosidase gene was selected due to its non-essential nature in *Leptospira* spp. and ease of evaluating a mutant’s phenotype^16^.

However, no colonies could be recovered after transformation with plasmids containing both Cas9 and sgRNA, even though a large number of colonies were obtained when the Cas9-plasmid alone was utilized. These results led us to conclude that as in most prokaryotic cells^64,65^, DSBs are lethal to *L. biflexa* cells, indicating the lack of NHEJ machinery.

In bacteria, the NHEJ system, when present, is carried out by that homologous to the eukaryotic Ku protein and ATP-dependent DNA ligase^66–68^. Only a few bacteria have annotated genes coding for Ku and the ATP-dependent LigD proteins, including the human pathogens *Mycobacterium tuberculosis, Pseudomonas aeruginosa*, and *Bacillus anthracis*^69^. Some bacterial species, such as *Clostridium cellulolyticum*, are killed by genomic DSBs generated by Cas9 cleavage despite the presence of NHEJ systems^70^. The NHEJ system was not found in the *Leptospira* genomes.

To overcome DSB lethality in some bacteria and achieve gene silencing rather than disruption, a newer variant of the CRISPR/Cas9 technology has been developed. CRISPRi consists in a catalytically “dead” Cas9 (dCas9) with a point mutation within the nuclease domains^18,38^. This protein, when interacting with sgRNA, is capable of binding to genomic loci causing RNA polymerase steric hindrance, blocking transcription initiation, if bound to the promoter, or elongation, if bound to the CDS.

We adapted the dCas9 methodology to *L. biflexa* cells, by constructing plasmids expressing dCas9 alone or along with different sgRNA, aiming at hybridization to the coding and template strand of DNA. Zhao *et al*.^38^, using the same strategy to obtain gene silencing in *S. aureus*, demonstrated almost complete gene silencing when sgRNA paired with the coding strand, in contrast to a slight reduction in phenotype when pairing with the sgRNA template strand.

In contrast to the lethality of the CRISPR/Cas9 system, *L. biflexa* cells could survive sgRNA expression along with dCas9, corroborating the incapability of DSB repair in the absence of a template DNA and suggesting that no off-target in essential genes occurred with the sgRNAs employed. When dCas9 was bound to the β-galactosidase gene and base pairing occurred between sgRNA and the coding strand, promising results were obtained: a complete gene silencing was observed, as assessed by enzyme assays and real-time PCR, confirming that indeed, dCas9 bound to the target gene hampered RNA polymerase elongation. CRISPRi has been successfully utilized in other microorganisms^39,42,71–74^. Modulating the degree of gene silencing, depending on the DNA strand to which sgRNA hybridizes, could be a valuable feature since a gradient of a protein expressed in bacterial cells could render distinct phenotypes, facilitating the validation of the protein function.

CRISPRi tool could also render satisfactory results when applied for highly expressed genes, e.g., *dnaK*. Interestingly, when we targeted another leptospiral gene, *dnaK*, no colonies could be recovered when sgRNA designed to hybridize to the coding strand was used, a condition in which complete gene silencing was expected. These data led us to suggest the essential role of DnaK protein in *L. biflexa*. Accordingly, random transposon libraries in *Leptospira* failed to recover DnaK-deficient strains^9,10^, strengthening the necessity of this gene. Furthermore, in several other bacteria, DnaK is essential for cell growth not only at elevated temperatures but also under optimal conditions, although the reasons for its crucial role are still unclear^75^.

Flagellar motor switch *fliG2* gene knockout by homologous recombination in *B. burgdorferi* rendered a reduced mobility phenotype^51^, longer time required for colony formation and smaller colonies. *L. biflexa* possess an annotated gene (LEPBI_RS16770) coding for a protein with 70% similarity to borrelial FliG2. CRISPRi gene silencing drastically reduced protein expression, which was accompanied by colonies with reduced diameter, implying decreased motility. It is the first demonstration that FliG protein is required for leptospiral motility.

Considering the episomal nature of the system, the generation of scape mutants could be an issue when applied to pathogenic strain for virulence factor validation, due to host selective pressure; nevertheless, the potential role of LigA and LigB protein in leptospiral virulence was demonstrated by the expression of episomal TALEs utilizing the same backbone (pMaOri)^16^ and, by our results, gene silencing is maintained when leptospires are grown in media with no plasmid selection. Because of pMaOri backbone, plasmids obtained in this work could be used to transform *Leptospira* spp. and currently, we are trying to establish this methodology in the pathogenic strain.

In conclusion, we report for the first time, the successful application of the recent CRISPRi methodology in *Leptospira*, expanding the genetic tools for target mutagenesis in these microorganisms. It is anticipated that the application of this one-step knockdown system to pathogenic strains will greatly advance our knowledge of the basic biology and virulent mechanisms of leptospires.

## Supplementary information

Dataset 1
