# Supplementary material for: Gene silencing based on RNA-guided catalytically inactive Cas9 (dCas9): a new tool for genetic engineering in Leptospira
Source: Sci Rep. 2019 Feb 12;9:1839. doi: 10.1038/s41598-018-37949-x (PMC6372684; doi:10.1038/s41598-018-37949-x)
Supplement: Supplementary file 1 — Dataset 1 [file 41598_2018_37949_MOESM1_ESM.pdf]

Supplementary figures for:

Gene silencing based on RNA-guided catalytically inactive Cas9 (dCas9): a new tool for genetic engineering in *Leptospira*

Fernandes, LGV[1*]; Guaman, LP[2]; Vasconcellos SA[3]; Marcos B. Heinemann, MB[3]; Picardeau[4], M and Nascimento, ALTO[1*]

[1]Laboratório Especial de Desenvolvimento de Vacinas, Instituto Butantan, Avenida Vital Brasil, 1500, 05503-900, Sao Paulo, SP, Brazil; [2]Universidad Tecnológica Equinoccial, Centro de Investigación Biomédica, Facultad de Ciencias de la Salud Eugenio Espejo, Avenida Mariscal Sucre y Mariana de Jesús. Campus Occidental, 170105, Quito, Ecuador; [3]Laboratório de Zoonoses Bacterianas do VPS, Faculdade de Medicina Veterinária e Zootecnia, USP, Avenida Prof. Dr. Orlando Marques de Paiva, 87, 05508-270, Sao Paulo, SP, Brazil; [4] Institut Pasteur, Biology of Spirochetes Unit, 25 rue du Dr Roux, 75723, Paris, France;

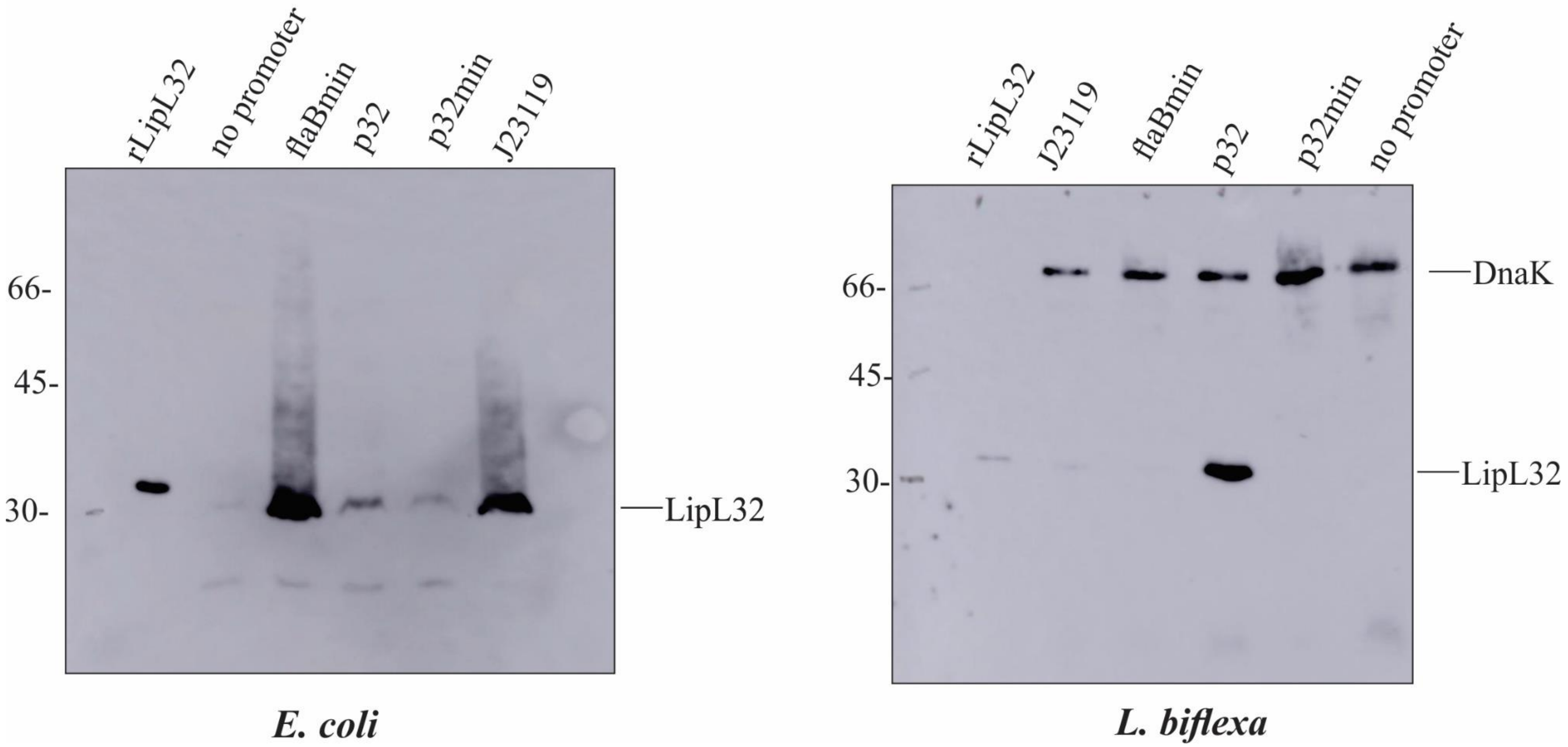

Supplementary Figure S1. Detection of LipL32 in recombinant *E. coli* and *L. biflexa*. Normalized extracts from recombinant *E. coli* and *L. biflexa* cells containing different constructions were evaluated by western blotting with anti-LipL32 antiserum for detecting heterologous LipL32 expression. Anti-DnaK antiserum was employed as a loading control for *L. biflexa* cell extracts and recombinant LipL32 (rLipL32) was employed as positive control.

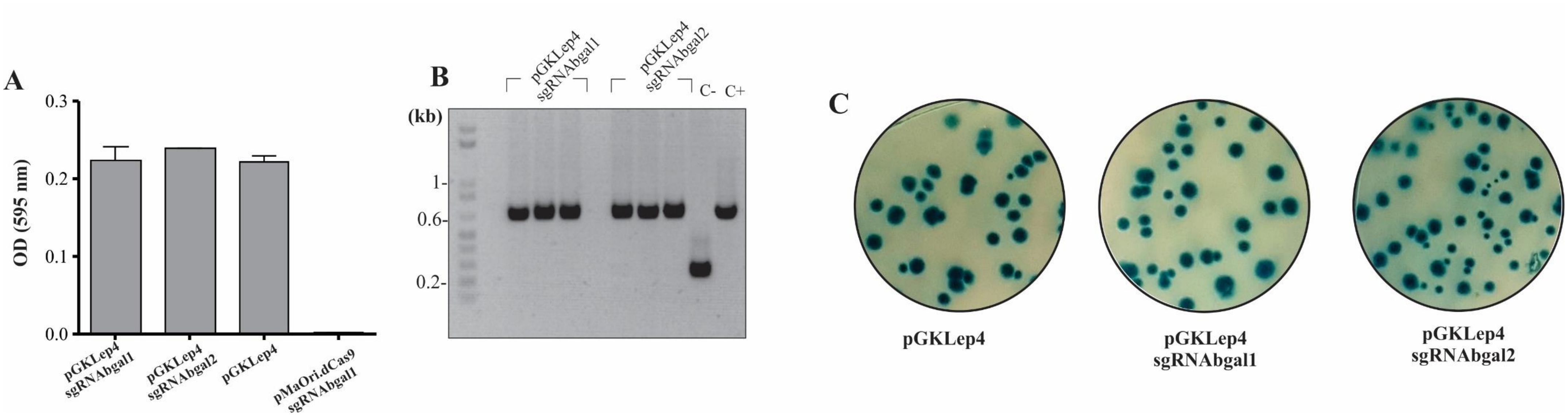

Supplementary Figure S2. β-galactosidase activity displayed by *L. biflexa* cells expressing sgRNA alone. *L. biflexa* cells were transformed with plasmids pGKLep4 or pGKLep4 containing the sgRNA cassettes, either sgRNAbgal1 or sgRNAbgal2. Three colonies from each plate were selected, grown in EMJH plus kanamycin and evaluated regarding β-galactosidase activity with X-gal substrate **(A)**. Cells containing pMaOri.dCas9sgRNAbgal1 were employed as control of complete gene silencing and graphics show the average of densitometric reading at 595 nm plus standard deviation of 3 biological replicates. **(B)** Confirmation of transformants and sgRNA cassette was performed by PCR with flanking primers. “C-” and “C+” refers to amplification of purified plasmids pGKLep4 and pGKLep4sgRNAbgal1, respectively. **(C)** X-gal solution was spread onto EMJH plates containing grown colonies.
